# Why follow-up matters in survival analysis: comparing cox proportional hazard regression and random survival forest for predicting heart failure outcomes

**DOI:** 10.1186/s12872-025-05165-x

**Published:** 2025-09-26

**Authors:** Emrah Gökay ÖZGÜR, Gülnaz Nural BEKİROĞLU

**Affiliations:** Department of Biostatistics, School of Medicine, Marmara University, Istanbul, Türkiye

**Keywords:** Person-time follow up rate, Random survival forest, Machine learning, Heart failure, Survival analysis

## Abstract

**Background:**

Heart failure (HF) remains a major global health burden with high mortality rates. Accurate survival prediction is essential for clinical decision-making. This study investigates how follow-up adequacy, quantified by Person-Time Follow-up Rate (PTFR), impacts the performance of survival models—specifically, Cox proportional hazards regression(CPHR) and Random Survival Forest (RSF).

**Methods:**

A routinely collected health dataset of 299 HF patients was analyzed. PTFR was calculated using the formal method by Xue et al. (2022), resulting in a PTFR of 45.6%. A simulated version of the dataset was generated by proportionally extending follow-up times to increase PTFR to 67.2%. Both CPHR and RSF models were applied to the original and simulated datasets. Model performance was assessed using C-index and Area Under Curve(AUC).

**Results:**

In the original dataset, the CPHR model achieved a C-index of 0.754 and AUC of 0.959, while the RSF model achieved a C-index of 0.884 and AUC of 0.988. In the simulated dataset, model performance improved slightly, with the improvement being more pronounced in RSF. It also more effectively identified clinically relevant predictors such as ejection fraction and serum creatinine. Increased PTFR led to better model stability and predictive accuracy.

**Conclusion:**

Improving PTFR enhances the validity and robustness of survival models. RSF outperformed Cox regression across both datasets, particularly under higher PTFR. Strategies such as extending follow-up duration and integrating data sources can help increase PTFR. These findings underscore the importance of adequate follow-up in predictive modeling and support the use of machine learning in clinical survival analysis.

## Background

Cardiovascular diseases (CVDs) remain the leading cause of death worldwide, accounting for approximately 17.9 million deaths annually and representing 31% of all global deaths [1]. Among these, heart failure (HF) is a complex clinical syndrome in which the heart is unable to pump blood sufficiently to meet the metabolic demands of the body, often developing as a consequence of underlying conditions such as ischemic heart disease, hypertension, or diabetes [2, 3]. HF is typically classified into two main subtypes based on ejection fraction (EF): heart failure with reduced EF (HFrEF; EF < 40%) and heart failure with preserved EF (HFpEF; EF ≥ 50%) [4–6]. These subtypes differ in clinical course, treatment response, and prognosis. While tools such as the New York Heart Association (NYHA) functional classification are widely used to assess disease severity, their subjectivity often limits predictive accuracy [7, 8]. The clinical trajectory of HF is highly heterogeneous, complicating decision-making and underscoring the importance of early-stage risk stratification [9].

This complexity makes not only model selection but also data quality—particularly follow-up adequacy—critical. In survival analysis, the Person-Time Follow-up Rate (PTFR) quantifies the proportion of potential follow-up time that is actually observed and has a direct impact on the accuracy of estimates [10]. Recent methodological research has shown that low PTFR can lead to both underestimation and overestimation of event probabilities, depending on censoring patterns, event rates, and follow-up length [11, 12]. Liu et al. [12] reported that insufficient follow-up in early-phase clinical studies often results in underestimation of event probabilities, whereas Alligon et al. [13] highlighted risks of both under- and overestimation. Furthermore, evidence from multimorbidity and frailty research emphasizes that follow-up duration is critical for accurately capturing disease trajectories. For example, Halder et al. [14], using Longitudinal Aging Study in India (LASI) data, demonstrated a strong association between multimorbidity and frailty, stressing that inadequate follow-up could bias such associations. Similarly, Halder et al. [15] examined the relationship between frailty and non-laboratory-based cardiovascular risk scores, noting that follow-up length significantly influences the accuracy of predictive models.

The importance of PTFR extends beyond statistical considerations to clinical and policy implications. Although PTFR levels of ≥ 60% have been recommended in the literature to enhance model reliability [11], such thresholds are rarely reported in applied studies, and their effect on model performance has been infrequently investigated. In high-mortality conditions such as HF, failure to account for PTFR may lead to flawed clinical predictions and misguided policy decisions.

In recent years, machine learning (ML)–based survival models have demonstrated superior predictive performance compared to traditional methods due to their ability to capture nonlinear relationships and high-order interactions [16–18]. Among these, RSF have shown particular advantages over the Cox Proportional Hazards Regression (CPHR) model in handling complex data structures, processing a large number of variables simultaneously, and maintaining robustness under high censoring rates [19, 20]. RSF also enables variable importance ranking, interaction modeling, and flexible handling of nonlinear effects, making it especially suitable for examining how follow-up adequacy impacts model performance. These properties were key factors in selecting RSF as the ML algorithm for this study. While deep learning–based methods (e.g., DeepSurv, DeepHit) have shown promise, they typically require much larger datasets and substantial computational resources [21, 22]. Some HF-specific studies (e.g [23])., have approached survival as a classification problem without incorporating time-to-event or censoring information, limiting the understanding of how follow-up adequacy influences model performance.

This study tests the hypothesis that follow-up adequacy, measured by PTFR, is a key determinant of predictive accuracy in survival modeling. We calculated PTFR for a routinely collected HF dataset and analyzed it using both CPHR and RSF models. We then created a simulated cohort exceeding the 60% PTFR threshold recommended in the literature and re-evaluated both models. By comparing results across datasets, we aimed to systematically assess the influence of PTFR on model accuracy and the reliability of clinical risk predictions.

## Data and methods

### Dataset

In this study, we utilized the “Heart Failure Clinical Records Dataset” which contains clinical data of heart failure patients collected in 2015 at the Faisalabad Institute of Cardiology and Allied Hospital in Punjab, Pakistan. The dataset is publicly available on the Kaggle platform (https://www.kaggle.com/datasets/andrewmvd/heart-failure-clinical-data*).* It includes demographic and clinical information from 299 patients, aged between 40 and 95 years. The dataset comprises 12 variables including age, sex, ejection fraction, platelet, serum sodium, diabetes, anemia, serum creatinine, creatinine phosphokinase, high blood pressure, smoking status and event status. In addition, survival status and follow-up times (measured in days) are recorded.

### PTFR calculation

To rigorously assess the adequacy and quality of follow-up in the dataset, we applied the Formal PTFR method as proposed by Xiaonan Xue et al. (2022) [11]. Unlike simple ratio methods, this formal approach accounts for censoring and event times using survival function estimates and interval censoring techniques.

The formal PTFR is defined as the ratio of the observed person-time to the expected person-time assuming no dropouts, expressed as a percentage:

$$\:{{\upeta\:}}_{PTFR}=\frac{\sum\:_{i=1}^{N}\text{m}\text{i}\text{n}({T}_{i},{C}_{i},{\uptau\:})}{\sum\:_{i=1}^{N}\text{m}\text{i}\text{n}({T}_{i},{\uptau\:})}$$

where:

- $\:{T}_{i}$ is the event time for individual iii,
- $\:{C}_{i}$ is the censoring time for individual iii,
- $\:{\uptau\:}$ is the maximum follow-up time (study end time),
- N is the total number of patients.

Because event times for dropouts are not directly observed, the method estimates the survival function and expected event counts via nonparametric maximum likelihood estimation (NPMLE) techniques, which are appropriate for interval-censored data. This enables a more accurate estimation of the maximum potential person-time follow-up. In the literature, a PTFR value of at least 60% is generally recommended to ensure sufficient follow-up for reliable survival modeling [11].

In the current analysis, the maximum follow-up time was set at 285 days for the original dataset, and the formal PTFR was computed accordingly. This approach allowed a robust quantification of follow-up completeness, yielding a PTFR of approximately 45.6% in the original dataset.

### Simulation of a dataset with increased PTFR

In the original dataset, the PTFR was calculated as approximately 45.6%, indicating relatively short follow-up durations that could limit the reliability of model performance metrics. To overcome this limitation and enable more robust model comparisons, a simulated dataset with a higher PTFR was generated.

The simulation was performed using the R programming language without relying on any external packages. Specifically, the approach involved rescaling the follow-up time in the original dataset by a constant multiplier.

The simulation steps were as follows:

1. The total person-time in the original dataset was calculated to determine the current PTFR.
2. Based on the desired PTFR, the target total person-time was computed.
3. To reach the target PTFR, each subject’s follow-up time was multiplied by a suitable scaling factor. This process increased the follow-up duration while keeping the event status and other covariates unchanged.
4. As a result, the overall structure and distribution of the data were preserved, but the dataset reflected a more complete follow-up scenario.

The final simulated dataset achieved a PTFR of 67.2%, offering improved follow-up completeness and a more appropriate basis for comparing model performance.

### Survival analysis methods

Two survival analysis techniques were applied to both the original and simulated datasets:

- CPHR: A semi-parametric model evaluating the effects of covariates on survival under the proportional hazards assumption. Kaplan-Meier survival curves were plotted for visualization, and log-rank tests were used to compare groups.
- RSF: A non-parametric, machine learning method based on ensemble decision trees that models complex, nonlinear effects without proportional hazards assumptions. The datasets were split into 70% training and 30% testing subsets for RSF modeling.

### Model performance evaluation

Model performances were evaluated using the Concordance index (C-index), Standard Error (SE), and Area Under the Curve (AUC), allowing a comparative assessment of predictive accuracy between the original and simulated datasets. The C-index measures the model’s ability to correctly rank patients according to their survival risk (global discrimination), while the time-dependent AUC assesses classification performance at specific time points. Using both metrics provides a more comprehensive evaluation of model performance.

### Software and packages

All analyses were performed using R version 4.4.1 (R Core Team, 2024) and RStudio 2024.09.0 Build 375. The key R packages used include survival, survAUC, timeROC, randomForestSRC, survcomp, pec, caret, pROC, simsurv, and interval for PTFR calculation.

## Results

This study evaluated the performance of CPHR and RSF models using both the original and simulated datasets. The simulation was specifically designed to increase the PTFR, allowing for the examination of how improved follow-up adequacy influences model performance, discriminatory power, and variable stability.

### Model performance

#### CPHR

The proportional hazards assumption was tested using Schoenfeld residuals and was satisfied in both the original (*p* = 0.39) and simulated (*p* = 0.43) datasets.

**Table 1:** CPHR results for original and simulated datasets

Variable	HR (Original)	95% CI (Original)	*p*-value (Original)	HR (Simulated)	95% CI (Simulated)	*p*-value (Simulated)
Age	1.048	1.025–1.072	< 0.001	1.044	1.021–1.068	< 0.001
Ejection Fraction	0.947	0.924–0.970	< 0.001	0.944	0.920–0.967	< 0.001
Serum Creatinine	1.487	1.215–1.820	< 0.001	1.429	1.185–1.724	< 0.001
Anemia	1.609	1.118–2.315	0.010	1.589	1.115–2.265	0.011
Creatine Phosphokinase	1.000	1.000–1.000	0.048	1.000	1.000–1.000	0.050
High Blood Pressure	1.547	1.093–2.190	0.014	1.502	1.062–2.124	0.022

In the original dataset, the CPHR model yielded a C-index of 0.754 (SE = 0.0261) and an AUC of 0.959, indicating high discriminatory and classification performance. Statistically significant variables included in order of importance age (*p* < 0.001), ejection fraction (*p* < 0.001), serum creatinine (*p* < 0.001), anemia (*p* = 0.015), creatinine phosphokinase (*p* = 0.026), and high blood pressure (*p* = 0.048) (Table 1).

In the simulated dataset, where PTFR increased from 45.6 to 67.2%, the CPHR model showed similar discrimination with a C-index of 0.756 (SE = 0.0258) but a reduced AUC of 0.770, suggesting lower classification accuracy. Nevertheless, the same variables remained statistically significant: age (*p* < 0.001), ejection fraction (*p* < 0.001), serum creatinine (*p* < 0.001), anemia (*p* = 0.018), creatinine phosphokinase (*p* = 0.033), and high blood pressure (*p* = 0.041).

**Fig. 1:**
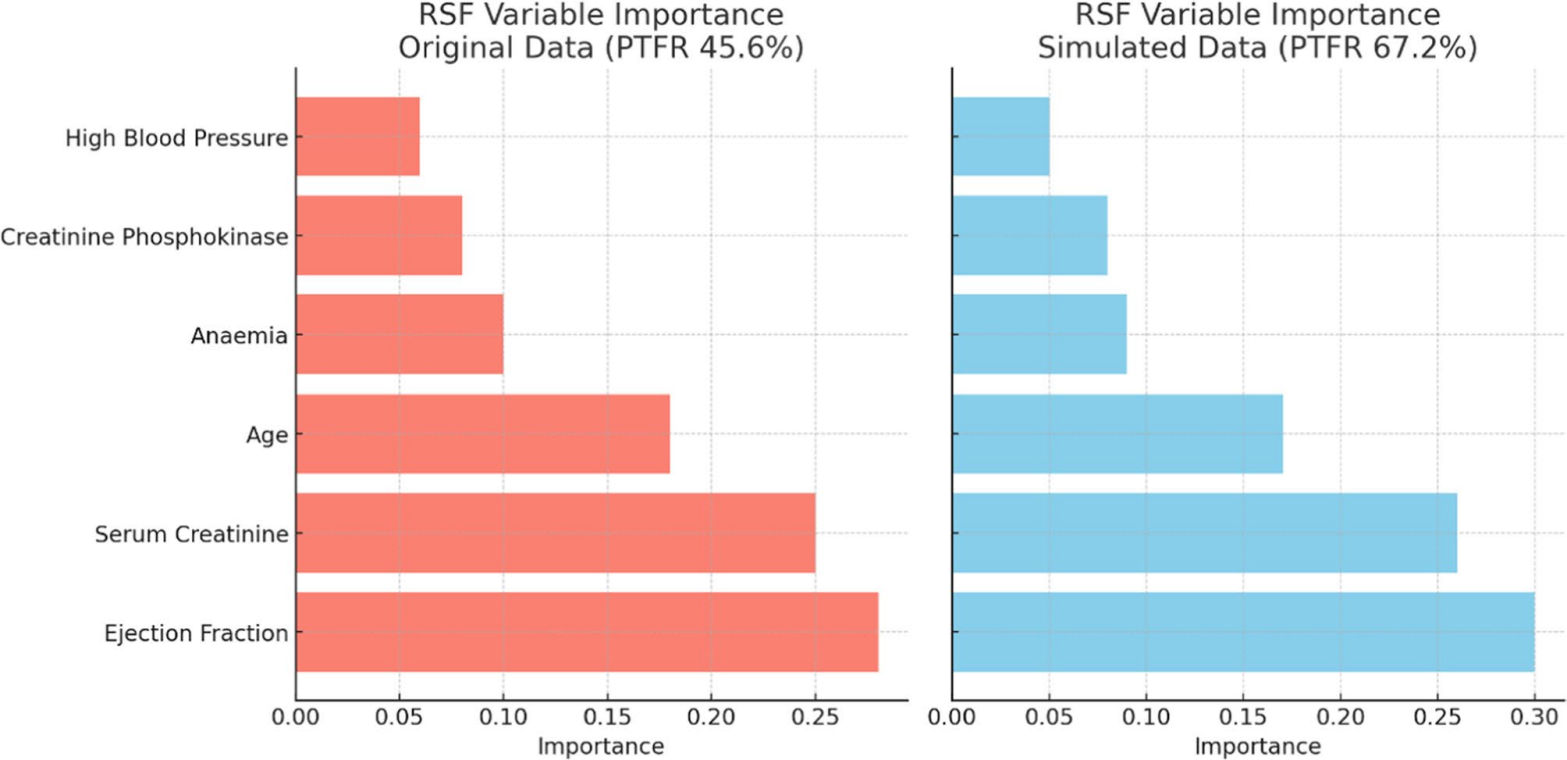
RSF variable importance of original and simulated data

Figure 1 depicts Kaplan–Meier survival curves comparing the original dataset (PTFR 45.6%) with the simulated dataset (PTFR 67.2%). The log-rank test indicated a statistically significant difference between the two curves (*p* = 0.0073). The simulated dataset consistently exhibited higher survival probabilities across the follow-up period, particularly at mid-to-late time points. This improvement reflects the effect of increased PTFR in retaining a larger proportion of patients at risk, as shown in the number-at-risk table. In contrast, the original dataset, characterized by lower PTFR, demonstrated a steeper decline in survival probability and a faster drop in the number of patients under observation.

#### RSF model

The RSF model generally demonstrated favorable performance compared to the CPHR model. For the original dataset, it achieved a C-index of 0.884 and an AUC of 0.988. In the simulated dataset with improved PTFR, the C-index slightly increased to 0.890, while the AUC declined to 0.862. These results suggest that while RSF maintained good discriminatory capacity, its performance advantage was not consistent across scenarios. This underscores the need to interpret RSF’s superiority cautiously, as it may depend on data characteristics and follow-up quality.

**Fig. 2:**
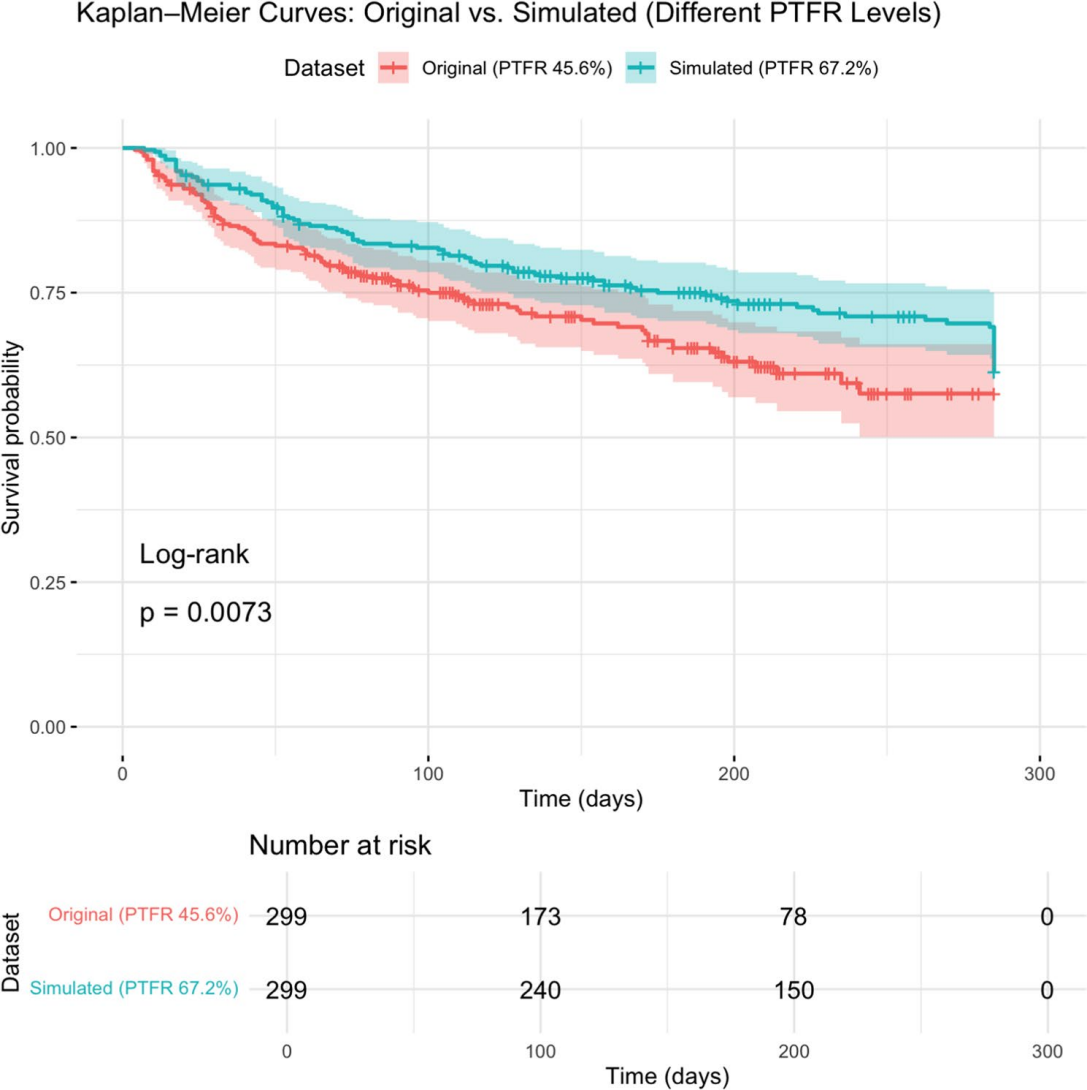
Kaplan-meier curves of original and simulated data

Figure 2 presents a side-by-side comparison of RSF variable importance rankings for the original dataset (PTFR 45.6%) and the simulated dataset (PTFR 67.2%). In both datasets, ejection fraction, serum creatinine, and age consistently emerged as the top three most important predictors. This consistency indicates that increasing PTFR did not alter the core set of predictive variables, but rather enhanced the stability and reliability of the model’s predictions.

Raising PTFR through simulation led to minor shifts in the importance rankings of mid-level predictors such as creatinine phosphokinase and serum sodium, while variables with low importance remained largely unaffected. 

**Fig. 3:**
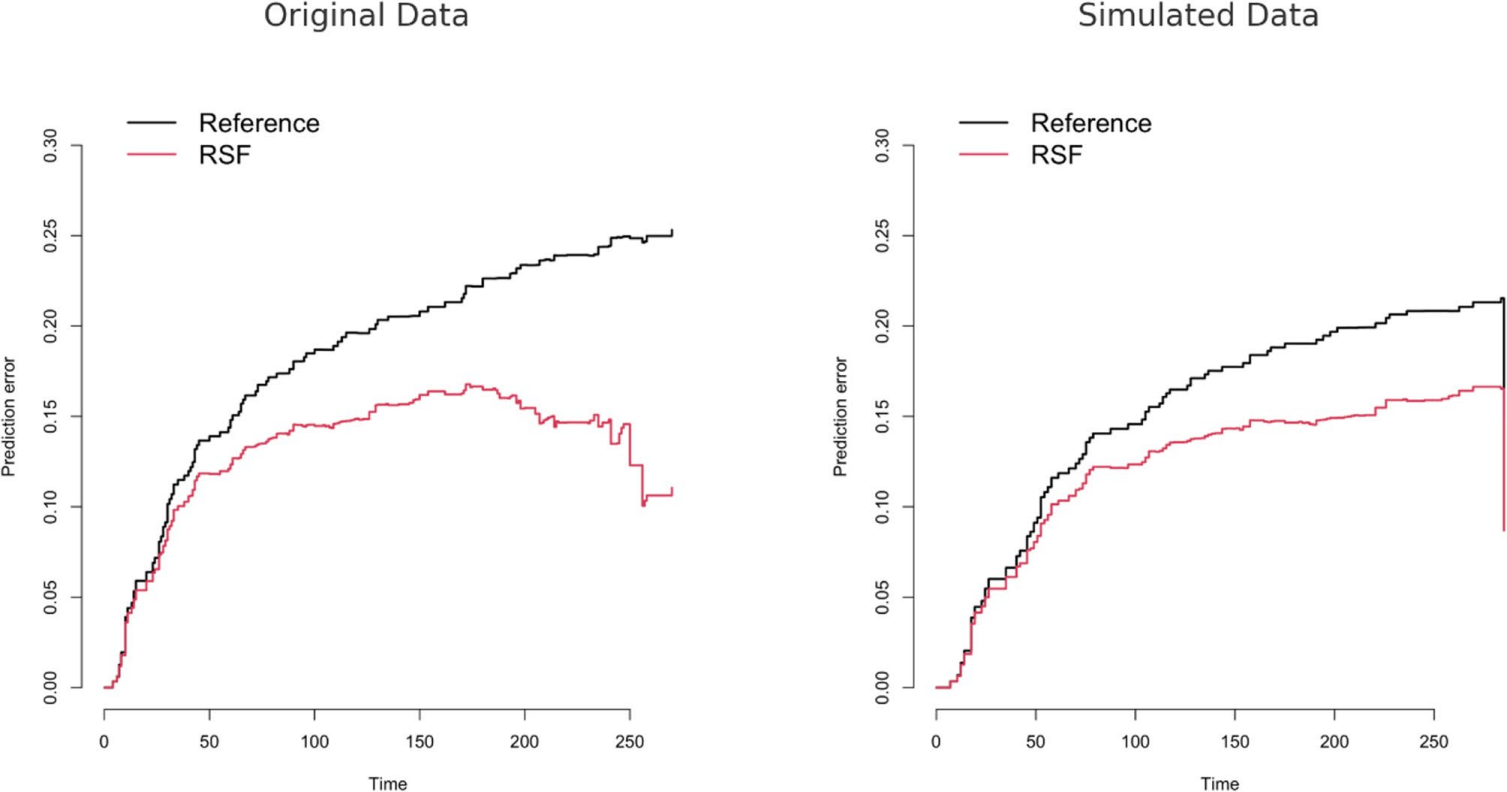
Time-dependent prediction error curves for RSF models trained on original (left) and simulated (right) datasets

In terms of model calibration, the RSF model demonstrated superior performance over the reference model (CPHR) in both the original and simulated datasets (Fig. 3).

- For the original dataset, the RSF model had an Apparent Error (AppErr) of 0.103 and a 0.632 bootstrap error of 0.133. In comparison, the reference model showed a higher AppErr of 0.188, and its 0.632 bootstrap error was approximated to 0.210.
- For the simulated dataset, the RSF model achieved an AppErr of 0.099 and a 0.632 bootstrap error of 0.131, while the reference model had an AppErr of 0.185 and an estimated 0.632 bootstrap error of 0.208.

These results indicate that the RSF model consistently yielded lower error rates, highlighting its superior calibration performance compared to the CPHR model. Moreover, the improvement in follow-up (as indicated by increased PTFR in the simulated data) was also associated with better model calibration.

### PTFR impact

The PTFR increased from 45.6% in the original dataset to 67.2% in the simulated dataset, indicating improved follow-up adequacy. This change contributed to marginally more stable performance metrics overall, though not uniformly across all model types or evaluation criteria.

#### Comparison of predictors

The CPHR model identified consistent statistically significant predictors in both original and simulated datasets, namely:

- age,
- ejection fraction,
- serum creatinine,
- anemia,
- creatinine phosphokinase, and.
- high blood pressure (Table 1).

On the other hand, the RSF model consistently ranked the following variables as the most important in both datasets:

- ejection fraction,
- serum creatinine,
- age,
- creatinine phosphokinase,
- serum sodium, and.
- platelet count (Table 2).

**Table 2:** Model comparison: CPHR vs. RSF

Model	Dataset	C-index	SE	AUC	PTFR	Significant/Important Variables
CPHR	Original	0.754	0.0261	0.959	45.6%	age, ejection fraction, serum creatinine, anemia, CPK, high blood pressure
CPHR	Simulated	0.756	0.0258	0.770	67.2%	age, ejection fraction, serum creatinine, anemia, CPK, high blood pressure
RSF	Original	0.884	0.0100	0.988	45.6%	ejection fraction, serum creatinine, age, CPK, serum sodium, platelet
RSF	Simulated	0.890	0.0009	0.862	67.2%	ejection fraction, serum creatinine, age, CPK, serum sodium, platelet

These findings suggest that both models were able to detect clinically relevant and stable variables, while RSF exhibited superior predictive ability. Moreover, improved follow-up duration positively impacted the robustness and consistency of variable selection.

Table 2.

## Discussion

In this study, we compared the classical CPHR model with the ML -based RSF model to predict survival in HF patients. This comparison was conducted using both real and simulated datasets, with a particular focus on the PTFR, a key indicator of follow-up quality. While the original dataset had a PTFR of 45.6%, the simulated dataset achieved a PTFR of 67.2%. This increase in PTFR led to an overall improvement in model performance, which was more pronounced in the RSF model.

Our findings strongly reinforce the conclusions of Xue et al. [11], who demonstrated that inadequate PTFR not only introduces systematic bias into survival estimates but also substantially compromises the power, stability, and reliability of predictive models. They highlighted that models built on datasets with PTFR below 60% experienced notable reductions in both statistical power and predictive accuracy. In our study, although increasing PTFR to 67.2% through simulation did not alter the set of statistically significant predictors, it led to enhanced model stability and discrimination. This suggests that even when covariate significance remains unchanged, higher PTFR contributes to increased confidence in model predictions—an essential aspect of clinical risk modeling. Similarly, Liu et al. [12] reported that in early-phase clinical trials, insufficient follow-up time increases the risk of underestimating event probabilities. However, depending on censoring patterns and outcome definitions, inadequate follow-up may also result in overestimation of survival or event probabilities, especially when competing risks or left truncation are ignored [13]. These issues introduce bias by excluding early events or by misclassifying competing outcomes as censored observations. These observations align with our findings, indicating that increasing PTFR enhances model robustness and reduces both under- and over-estimation risks in both statistical and ML based approaches. These findings also underscore the practical value of simulation-based PTFR enhancement. In our analysis, the simulated dataset with PTFR raised above 60% (67.2%) reproduced the same set of statistically significant predictors as the original dataset. This consistency suggests that while the original dataset may suffer from limited predictive power due to low PTFR, the observed associations are still meaningful. Therefore, when working with survival datasets having PTFR below 60%, it is advisable to conduct supplementary analyses— such as PTFR-based simulations or follow-up completeness assessments—to validate the stability and interpretability of the results.

Moreover, the RSF model outperformed the CPHR model in both real and simulated settings, with the performance gap becoming more evident as PTFR increased. This finding is in line with previous studies by Ishwaran et al. [18] and Mogensen et al. [19], which showed that RSF effectively models nonlinear and high-dimensional survival data. Wang et al. [20] also reported that RSF provides more stable predictions in scenarios involving complex interactions and variable follow-up durations. In our study, the RSF model more clearly highlighted clinically relevant variables such as age, ejection fraction, and serum creatinine compared to the CPHR model.

However, it is noteworthy that while RSF showed stable or improved performance with increased PTFR, the AUC of the CPHR model declined from 0.959 to 0.770 in the simulated dataset. This unexpected trend might stem from changes in the event-time distribution, a shift in the case-mix introduced by simulation, or potential limitations of the regression-based method when handling simulated follow-up structures. Unlike tree-based models like RSF, CPHR may be more sensitive to subtle changes in time-to-event structure or censoring patterns, which can influence its discrimination performance. Therefore, while increased PTFR generally improves model robustness, its effect may vary depending on the statistical framework and underlying assumptions.

The dataset used in this study has previously been analyzed by Chicco and Jurman [23], but their work focused on a classification task rather than survival analysis. In their study, the time variable was treated as an independent predictor, and model performance was evaluated using metrics such as accuracy and Matthews Correlation Coefficient (MCC). In contrast, our analysis incorporated time-to-event and censoring information, making it methodologically distinct and more appropriate for survival modeling. Moreover, Chicco and Jurman used traditional ML classifiers like logistic regression and support vector machines, without applying time-to-event-specific models such as RSF or DeepSurv. Thus, despite using the same dataset, the methodological depth and time-to-event focus of our study provide a novel contribution to the literature.

Although deep learning–based survival models such as DeepSurv and DeepHit have shown strong performance in recent [21, 22], their application often requires large datasets and substantial computational power. RSF, on the other hand, offers a more accessible and interpretable solution for medium-sized datasets such as as ours.

From a public health perspective, ensuring adequate follow-up in longitudinal health databases is crucial for producing robust prognostic models that can guide clinical decision-making and inform health policy. In high-mortality conditions such as heart failure, inaccurate survival estimates resulting from inadequate follow-up can mislead resource allocation, patient counseling, and treatment prioritization. To address this, several strategies have been suggested in the literature to enhance PTFR values. Extending follow-up duration is one of the most direct methods, as longer observation periods naturally accumulate more person-time [11]. Minimizing loss to follow-up through robust tracking systems and patient retention strategies can further improve data completeness and effective follow-up time [24]. Refining inclusion criteria to prioritize patients likely to remain under observation—such as those with stable healthcare access—can enhance follow-up consistency [25]. From a data management perspective, integrating multiple data sources, including hospital records, insurance claims, and national registries, has been shown to boost PTFR by reducing right-censoring and improving longitudinal data quality [26]. Incorporating such measures into national health data quality frameworks, as supported by evidence from other chronic disease contexts [27, 28], can improve prediction accuracy, enhance model stability, and facilitate better-targeted public health interventions.

### Limitations

This study has some limitations that should be considered. First, the dataset was relatively small (*n* = 299) and originated from a single centre, which may limit the generalisability of the findings. Second, although rich in clinical variables, the dataset lacked information on treatment history, biomarker trajectories, and socioeconomic status, which may have limited the scope of interpretation. Third, the increase in PTFR was achieved through statistical simulation, which does not fully replicate the complexity of real-world follow-up dynamics such as non-random loss to follow-up or changes in clinical management over time. Fourth, external validation with independent datasets was not performed, and unmeasured confounding cannot be excluded.

### Future research directions

The findings of this study offer several concrete avenues for future research. First, to better elucidate the impact of varying PTFR levels, similar analyses could be conducted in patient populations beyond heart failure and across different disease types. Second, time-to-event artificial intelligence algorithms other than RSF could be explored to comparatively assess the effect of PTFR improvement on these models. Third, the feasibility and effectiveness of strategies aimed at increasing PTFR—such as extending follow-up duration, reducing loss to follow-up, and integrating multiple data sources—could be evaluated in real-world settings.

## Conclusion

This study contributes to the survival analysis literature in two significant ways. First, it highlights that the PTFR is not merely a measure of follow-up quality, but a critical factor that directly influences model accuracy, stability, and interpretability. Second, the RSF model exhibits superior predictive performance compared to the traditional CPHR model, particularly when follow-up adequacy is improved. Therefore, researchers and clinicians are encouraged to ensure sufficient follow-up time and person-time coverage in survival studies to generate more robust and reliable findings.

## Data Availability

The dataset used in the article is an open access dataset and can be accessed from this link. (https://www.kaggle.com/datasets/andrewmvd/heart-failure-clinical-data)

## Abbreviations

HF: Heart Failure
CVD: Cardiovascular Disease
EF: Ejection Fraction
NYHA: New York Heart Association
PTFR: Person-Time Follow-up Rate
ML: Machine Learning
CPHR: Cox Proportional Hazards Regression
AUC: Area Under The Curve
RSF: Random Survival Forest
NPMLE: Nonparametric Maxium Likelihood Estimation
C Index: Concordance Index
SE: Standart Error
MCC: Matthews Correlation Coefficient
